# The Association of Fasting Glucose, Insulin, and C-Peptide, with 19-Year Incidence of Coronary Heart Disease in Older Japanese-American Men; the Honolulu Heart Program

**DOI:** 10.3390/geriatrics3020022

**Published:** 2018-04-22

**Authors:** Nazneem Wahab, Randi Chen, Jess David Curb, Bradley J. Willcox, Beatriz L. Rodriguez

**Affiliations:** 1 CK Hui Heart Centre, Division of Cardiology, Royal Alexandra Hospital, University of Alberta, Rm 6S132A Robbins Pavillion, 10240 Kingsway Avenue, Edmonton, AB T5H 3V9, Canada; 2 Department of Research, Kuakini Medical Center, 347 N. Kuakini St, Honolulu, Hawaii, 96817, USA; rdc688a@gmail.com (R.C.); willcox@hawaii.edu (B.J.W.); 3 Department of Geriatric Medicine, John A. Burns School of Medicine, University of Hawaii, Hale Pulama Mau, 9th Floor, 347 N. Kuakini St, Honolulu, Hawaii, 96817, USA; brodrigu@hawaii.edu; 4 Pacific Health Research and Education Institute, 3375 Koapaka Street, Suite I-540, Honolulu, Hawaii 96819, USA; 5 Escuela deMedicina, Tecnologico deMonterrey, AvenidaMorones Prieto 3000, Monterrey, NL, 64710, Mexico

**Keywords:** diabetes mellitus, glucose, coronary disease, insulin, follow-up studies

## Abstract

The role of fasting glucose, insulin levels, and C-peptide in coronary heart disease (CHD) in non-diabetic individuals remains uncertain. We examined the association between fasting glucose, insulin and C-peptide with the long-term incidence of CHD in Japanese-American men. In 1980–1982, from a random sample of the Honolulu Heart Program men (*n* = 1378), aged 61–81 years, data on several CHD and metabolic risk factors were obtained to examine the relation of fasting glucose, insulin and C-peptide to 19-year CHD incidence. Age-adjusted incidence of CHD increased with increasing quintiles of glucose, insulin and C-peptide. Age-adjusted CHD rates in the glucose quintiles were 11.9, 11.6, 14.4, 18.1 and 24.1 per 1000 person-years (trend *p* < 0.001). In individual Cox models (lowest quintiles of glucose, insulin and C-peptide as reference) the relative risks (95% confidence interval) of CHD incidence for the glucose quintiles adjusting for age, smoking, hypertension, cholesterol, physical activity, and body mass index, were 0.9 (0.6–1.4), 1.2 (0.8–1.8), 1.4 (0.9–2.2), and 1.7 (1.1–2.6), respectively (trend *p* = 0.004). Insulin and C-peptide were not significantly associated with CHD on multivariate analysis. Fasting glucose remained the only significant predictor of increased CHD risk (*p* = 0.003) in a model combining all 3 metabolic variables. In this cohort, only fasting glucose independently predicts long-term incidence of CHD. Age-adjusted insulin and C-peptide levels were associated with CHD incidence, but after adjustment for other risk factors, do not independently predict CHD.

## 1. Introduction

Type 2 diabetes is associated with a high risk of ischemic heart disease. Impaired glucose tolerance (IGT) is also associated with progressive atherosclerosis, increased cardiovascular events and cardiovascular mortality [1,2,3]. A dose–response relationship of abnormal glucose metabolism to incident coronary heart disease (CHD), CHD mortality, and total mortality has been previously described in Japanese-American men [4].

Insulin resistance and fasting insulinemia are associated with increased incidence of major coronary events [5]. C-peptide is a byproduct of insulin production and is a gauge of how much insulin is produced in the body. It has a longer half-life and is an easier measure of average daily insulin secretion [6,7]. In a broader population of non-diabetic individuals, a negative correlation between serum C-peptide level and high density lipoprotein cholesterol (HDL-C)levels has been identified [8]. A correlation of C-peptide levels to elevated triglycerides and reduced HDL-C is also suggested in the elderly population [9]. C-peptide levels may also be associated with the severity of angiographic coronary artery disease [10].

The objective of this study was to examine the relationship of fasting glucose (FBG), insulin and C-peptide to the long-term risk for incident coronary heart disease in elderly Japanese-American men who were 61–81 years of age at the baseline exam in 1980–1982.

## 2. Materials and Methods

The Honolulu Heart Program (HHP) began in 1965, following a cohort of 8006 Japanese-American men who were then ages 45–65 and living in Oahu, Hawaii. The HHP has a longitudinal design, and the cohort has been followed for more than 40 years. The out-migration of this cohort has been less than 1 per 1000 per year [4]. Using a very thorough hospital surveillance system, all new cases of CHD were identified [11,12].

A random sample of the study was invited to participate in an examination conducted during 1980–1982. The sample of 1378 Japanese-American men were then between ages 61–81 [13,14]. At the time of analysis, up to 19 years of follow-up data for CHD incidence were available (1980–1999).

Cases of CHD were ascertained by a panel of HHP physicians using a standard protocol. A broad definition of coronary heart disease was used, including acute myocardial infarction, cause of death listed as CHD, silent myocardial infarction, angina, coronary artery bypass grafting, or percutaneous coronary intervention, electrocardiographic evidence of infarction in the follow-up exam, and sudden death within one hour of presentation with unknown cause of death. Participants with prevalent CHD, previously diagnosed diabetes mellitus, and those being treated for diabetes were excluded from the analysis leaving 1099 patients for evaluation.

Data on demographics, anthropometry, smoking history, alcohol consumption, physical activity index, body mass index (BMI), fasting total cholesterol, HDL-C, and triglycerides were gathered using standardized procedures [13,14]. After fasting for at least 12 h, blood samples were collected and plasma was frozen at minus seventy degrees Celsius. Plasma glucose, insulin, and C-peptide levels were measured. Insulin and C-peptide were quantified with ^125^I-labelled radioimmunoassays. All laboratory measurements were done at the Northwestern Lipid Laboratory, at the University of Washington.

### Statistical Analysis

Subjects were divided into 5 groups based on quintiles of fasting blood glucose, insulin, and C-peptide levels. Cox regression analyses were conducted with the glucose, insulin and C-peptide quintiles in separate models, adjusting for age and standard risk factors including hypertension, smoking history, alcohol consumption, physical activity, body mass index (BMI), fasting total cholesterol, HDL-C and triglycerides. Multivariate analysis including glucose, insulin and C-peptide values in the same model was also performed, adjusting for age and other risk factors.

## 3. Results

The baseline characteristics for the study subjects by glucose quintiles are detailed in Table 1. The mean age across all 5 quintiles was approximately 67 years. Quintile 5 had the highest total cholesterol and triglyceride levels, and the lowest HDL level. The body mass index (BMI) increased with each glucose quintile. Similarly, both mean systolic and diastolic blood pressure measurements increased with successive quintiles (*p* < 0.01). Conversely, the highest incidence of current smoking was seen in the lowest glucose quintile (*p* < 0.01), whereas past smoking history was similar across the groups.

The mean fasting blood glucose in the reference group was 94 mg/dL, with a range of 73–98 mg/dL. The mean fasting glucose in the 5th quintile was approximately 175 mg/dL, with a range from 120–417 mg/dL (Table 2).

**Figure 1:**
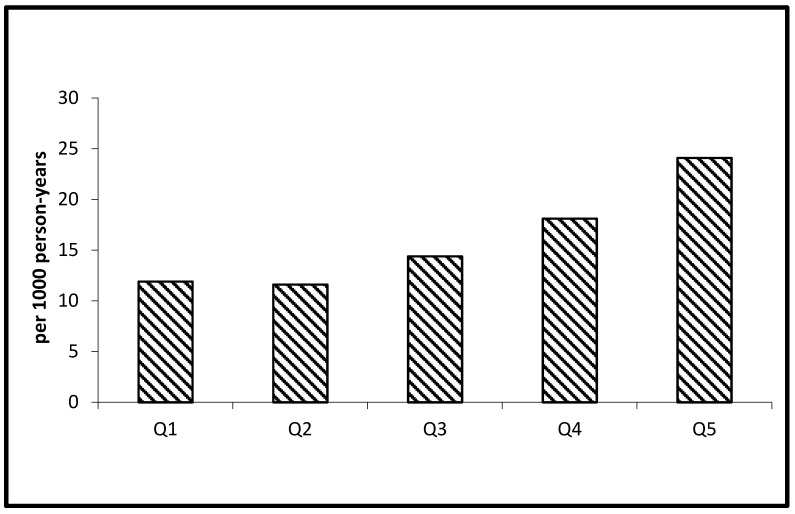
Age-adjusted incidence of CHD by glucose quintiles; trend test *p* < 0.001.

The age-adjusted incidence of coronary heart disease increased with increasing glucose quintile (Figure 1). The incidence in the lowest glucose quintile was 11.9 per 1000 person-years, increasing to 24.1 per 1000 person-years (trend test, *p* < 0.001) in the top quintile. When subjects with FBG ≥ 126 mg/dL at baseline were removed, adjusting for age, the trend test remained significant for increasing CHD risk (*p* = 0.04).

**Figure 2:**
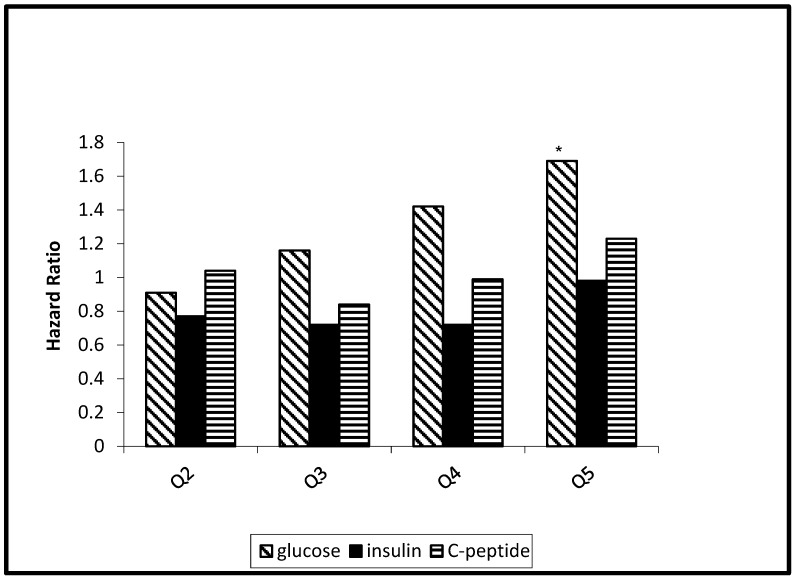
Risk of CHD by glucose, insulin and C-peptide quintiles from separate models. All models adjusted for age, hypertension, current and past smoking, total cholesterol, HDL-C, triglycerides, physical activity, alcohol consumption, body mass index; * *p* = 0.02. Test for trend adjusted for risk factors; glucose *p* = 0.004; insulin *p* = 0.96; C-peptide *p* = 0.47.

Multivariate analysis results including glucose, insulin and C-peptide quintiles are presented in separate models, using the first quintile as reference (Figure 2). In the glucose model, the highest incidence of CHD was seen in the top quintile (hazard ratio 1.7, *p* = 0.02). A significant trend for increasing incidence of CHD with increasing glucose quintile was observed after adjusting for other risk factors (*p* = 0.004).

Insulin and C-peptide showed a significant direct association with CHD risk when adjusting for age alone; however, there was no significant association in the multivariate analyses (Figure 2). Combined analysis with glucose, insulin and C-peptide values in the same model, demonstrated only glucose to be independently associated with 19-year incidence of CHD (*p* = 0.003).

Further analysis was done based on glucose groups defined by the current American Diabetes Association (ADA) definitions for normoglycemia (<100 mg/dL), impaired fasting glucose (100–125 mg/dL), and diabetes mellitus (≥126 mg/dL) [15]. In the multivariate analysis, the ‘diabetes’ group demonstrated a significant risk for CHD incidence (HR 1.8, *p* = 0.01), and for ‘impaired fasting glucose’, the hazard ratio was 1.18 (95% CI 0.84–1.66). Adjusting for age alone, a significant trend for increasing CHD risk with increasing glucose values was observed (*p* < 0.001). The trend test, when adjusting for other risk factors, remained statistically significant (*p* = 0.02). 

## 4. Discussion

Insulin is important for glucose, lipid and protein metabolism. It is postulated to increase myocardial blood flow in both ischemic and non-ischemic myocardial tissue in Type 2 diabetics [16]. Age-adjusted insulin values were predictive of CHD incidence over 19 years; however, in analyses adjusting for other risk factors, increasing insulin levels were not independently predictive of long-term risk for coronary heart disease.

C-peptide is a byproduct of insulin production [6,7]. It may also have a direct physiological role in attenuating secondary microvascular complications of diabetes [17,18]. However, C-peptide levels have also been associated with increased cardiovascular complications and overall death in non-diabetic adults [19,20]. Increased concentrations of insulin, C-peptide and leptin have been associated with increased intra-abdominal fat, in both the short and long term, in Japanese-Americans without diabetes [21]. Age-adjusted C-peptide was associated with CHD, but after adjustment for other risk factors, our analysis did not demonstrate an independently predictive capacity of C-peptide levels for the development of CHD after 19 years, in this cohort of Japanese-American men.

It is suggested that patients with CHD and abnormal glucose metabolism (impaired fasting glucose (IFG) or diabetes mellitus(DM) are at higher risk for long-term cardiac complications [22]. In patients with established CHD, incidental fasting hyperglycemia may increase their mortality risk beyond 2 years [23]. A trend toward increased angiographic evidence of coronary artery disease has been suggested in patients with mild, sub-clinical abnormalities of glucose metabolism [1,10]. Conversely, Lenzen et al. suggest that in at least the short term, newly diagnosed DM confers only a moderate risk for cardiovascular(CV)events, whereas IFG and IGT are not independent predictors [24]. Despite an association of elevated C-peptide levels with all-cause and cardiovascular mortality in younger adults without diabetes [20], in older adults, the mechanisms underlying the age-related increase in DM and related CHD remain uncertain [25]. This may be due to the heterogeneity of the population and the current lack of understanding of the biology of aging.

Among elderly men of Japanese ancestry, impaired fasting glucose, impaired glucose tolerance, and undiagnosed diabetes are highly prevalent [26]. Abnormal glucose metabolism in this population, at middle age, was also associated with increased risk for CHD incidence and total mortality [4], sudden cardiac death [27] and stroke [28]. Fujimoto et al. found visceral adiposity, blood pressure and plasma glucose to be risk factors for incident CHD over a 10-year period, in a population of 175 Japanese-American men, independent of a history of diabetes [29]. Of note, in this study, the 2-h post-load glucose values were not significant in multivariate analysis, when impaired fasting glucose was in the model. 

Our analysis, using fasting glucose at baseline, demonstrates a direct relationship with 19-year CHD incidence in middle-age and elderly Japanese-American men after risk factor adjustment, including BMI. These findings were consistent when analyses were conducted using fasting glucose quintiles, as well as the ADA definitions for IFG and diabetes [15]. A statistically significant trend (*p* < 0.05) was observed after risk factor adjustment for fasting glucose quintiles or when the ADA definitions were used to examine the association between fasting glucose levels and CHD incidence. In the model that included glucose, insulin, and C-peptide values, only fasting glucose remained significant in predicting long-term CHD risk. These results of fasting blood glucose are based on data when the HHP cohort men were aged 61–81 years at baseline. However, data from HHP when the men were 71–93 years old has shown that with advancing age, 2-hour oral glucose tolerance tests are increasingly important in the diagnosis of diabetes and as a predictor of cardiovascular disease(CVD) mortality in Japanese-American men [30].

In this cohort, it may be that fasting insulin and C-peptide are more closely linked to other variables included in the multivariate models and with the components of the metabolic syndrome, whereby not being independently associated with CHD once adjusted in the multivariate models. It is also possible that there is another mechanism playing a significant role in the association between fasting glucose and CHD incidence that is independent of the variables adjusted for in the models.

There is growing attention to the importance of studying ethnic minority groups for CVD outcomes, mechanisms of disease, risk factors, and for tailoring interventions. In North America, African Americans have a significantly higher risk of cardiovascular disease with a greater burden of CVD risk factors as compared to the non-Hispanic white population [31]. This may be due to the influence of multiple environmental, genetic and other factors. Conversely, studies of the Hispanic population compared with non-Hispanic whites have shown a significant association of Hispanic ethnicity with lower cardiovascular and all-cause mortality despite a higher prevalence of several cardiac risk factors in the Hispanic population [32,33]. South Asians in North America may have a higher prevalence of diabetes when compared to other ethnic minority populations not readily explained by traditional risk factors [34].

Significant variations in CVD risk factor burden and CVD prevalence are recognized in ethnic minorities in other regions as well. Patients of South Asian ethnicity living in the United Kingdom (UK) develop diabetes at an earlier age than Caucasian patients, despite having lower BMIs, and lower blood pressure. Compared with Caucasians with Type 2 DM, South Asians with Type 2 DM in the UK are at increased risk of CVD and present at a significantly younger age [35], whereas Chinese ethnicity conferred a lower risk of CVD in the UK, with the differences currently unexplained by known risk factors [36].

This research project is a prospective long-term cohort study, and therefore, one of the limitations is the possibility that not all confounding variables were controlled. This study was conducted on a Japanese-American male population; however, a case may be made about applicability of these results for other ethnic groups. Research has found that high glucose concentrations lead to higher CVD mortality in most regions of the world [37].

Fasting glucose was independently associated with CHD incidence in these analyses. Insulin and C-peptide levels were associated with CHD after adjusting for age but were not independently predictive of long-term coronary heart disease incidence after adjusting for other risk factors in this population. The findings of this investigation support the importance of impaired fasting glucose as a potential marker for increased CHD risk over nearly 20 years of follow-up in this population. These data suggest that the risk of fasting glucose on coronary heart disease incidence is nearly linear (test for trend for glucose, adjusted for risk factors *p* = 0.004), without a threshold value, and is independent of other established CHD risk factors. 

## Figures and Tables

**Table 1:** Baseline characteristics by glucose quintiles.

Variable	Q1 *	Q2 *	Q3 *	Q4 *	Q5 *	*p* Value
N	235	231	245	213	175	
Average Age (years)	67.46	67.87	67.62	67.5	67.96	
Total cholesterol (mg/dL)	205.4	209.8	215.1	214.2	214.8	<0.01
HDL-C ^†^ (mg/dL)	50.28	48.61	49.04	47.79	43.30	<0.01
Triglycerides (mg/dL)	133.8	146.5	163.6	176.1	257.8	<0.01
Treatment for dyslipidemia (%)	1	1	2	1	0	0.45
Body mass index (Kg/m^2^)	22.56	23.03	23.65	23.93	24.79	<0.01
Mean systolic blood pressure (mmHg)	134.4	136.9	138.8	141.5	143.3	<0.01
Mean diastolic blood pressure (mmHg)	79.91	80	81.12	82.85	83.57	<0.01
History or treatment of hypertension (%)	23	35	35	48	46	<0.01
Current Smoking (%)	26	22	22	14	17	<0.01
Past Smoking (%)	40	44	45	45	49	0.08
Alcohol (mL/week)	79.54	98.76	113.84	104.68	128.33	<0.01

* Q1, Q2, Q3, Q4, Q5 refer to quintile groups 1 to 5 respectively; ^†^ HDL-C; high density lipoprotein cholesterol.

**Table 2:** Mean value of glucose, insulin and C-peptide at each quintile of glucose.

Variable	Q1 *	Q2 *	Q3 *	Q4 *	Q5 *
Glucose (mg/dL)	94.12	101.18	106.4	113.46	143.28
Glucose range (mg/dL)	73.00–98.00	99.00–103.00	104.00–109.00	110.00–119.00	120.00–417.00
Insulin (µU/mL)	5.65	8.74	11.47	15.16	25.51
C-peptide (ng/mL)	0.76	1.16	1.54	1.93	2.91

* Q1, Q2, Q3, Q4, Q5 refer to quintile groups 1 to 5 respectively

## Abbreviation

CHD	Coronary heart disease
HHP	Honolulu Heart Program
IGT	Impaired glucose tolerance
DM	Diabetes mellitus
MI	Myocardial infarction
FBG	Fasting Glucose
BMI	Body mass index
HDL-C	HDL cholesterol
ADA	American Diabetes Association
Journal Subject Codes	3, 4, 190, 192
